# Risk of Cardiovascular Disease in Apnoeic Individuals: Role of Comorbid Insomnia Disorder

**DOI:** 10.3390/life12070944

**Published:** 2022-06-23

**Authors:** Matthieu Hein, Benjamin Wacquier, Jean-Pol Lanquart, Gwenolé Loas

**Affiliations:** Erasme Hospital, Department of Psychiatry and Sleep Laboratory, Université Libre de Bruxelles, ULB, Route de Lennik, 808, Anderlecht, 1070 Brussels, Belgium; benjamin.wacquier@erasme.ulb.ac.be (B.W.); jplanquart@skynet.be (J.-P.L.); gwenole.loas@erasme.ulb.ac.be (G.L.)

**Keywords:** cardiovascular risk, insomnia disorder, obstructive sleep apnoea syndrome, polysomnography

## Abstract

Given the limited data available, the aim of this study was to examine the 10-year cardiovascular disease (CVD) risk associated with comorbid insomnia disorder and its specific subtypes in apnoeic individuals. Data from 1104 apnoeic individuals recruited from the database of the Erasme Hospital Sleep Laboratory were analysed. Only apnoeic individuals with a Framingham Risk Score ≥10% were included in the group at moderate-to-high 10-year CVD risk. Logistic regression analyses were conducted to examine the risk of 10-year CVD risk associated with comorbid insomnia disorder and its specific subtypes in apnoeic individuals. Moderate-to-high 10-year CVD risk was present in 59.6% of the apnoeic individuals in our sample. After adjustment for the main confounding factors, multivariate logistic regression analyses revealed that comorbid insomnia disorder and, more particularly, its subtype with short sleep duration were significantly associated with moderate-to-high 10-year CVD risk in apnoeic individuals. In this study, we demonstrate that comorbid insomnia disorder and, more specifically, its subtype with short sleep duration appear to have a negative cumulative effect on 10-year CVD risk in apnoeic individuals, which justifies more systematic research and adequate therapeutic management of this disorder to allow for better cardiovascular disease prevention in this particular subpopulation.

## Highlights

- Apnoeic patients are a subpopulation at high risk of CVD.
- Comorbid insomnia disorder is associated with higher CVD risk in apnoeic patients.
- This association seems to be mediated by short sleep duration in apnoeic patients.
- Appropriate management of this disorder is essential in apnoeic patients.

## 1. Introduction

In the literature, there are many arguments in support of the association between obstructive sleep apnoea syndrome (OSAS) and cardiovascular disease (CVD). Indeed, the prevalence of OSAS may reach 70.0% in individuals with CVD, and the incidence of CVD is high in apnoeic individuals [1,2]. In addition, OSAS is associated with a negative impact on cardiovascular prognosis, both in the general population and in individuals with CVD [3,4,5]. Pathophysiologically, this higher risk of CVD in apnoeic individuals seems to be mediated by some deleterious mechanisms induced by intermittent hypoxia related to obstructive events (hyperactivation of the sympathetic nervous system, alterations in endothelial function, activation of pro-inflammatory pathways, alterations in the renin–angiotensin system, and metabolic dysregulations) [6]. In addition, in apnoeic individuals, there seems to be a severity-dependent effect of OSAS on the occurrence of these deleterious mechanisms induced by intermittent hypoxia [7]. However, despite a potential beneficial effect of OSAS treatments on these pathophysiological mechanisms that negatively impact cardiovascular prognosis [8], OSAS treatments have been shown to have only a limited effect on reducing cardiovascular risk in apnoeic individuals [9,10]. Thus, it seems necessary to carry out additional investigations in order to identify the potential cofactors involved in this higher risk of CVD in apnoeic individuals.

In apnoeic individuals, insomnia disorder is a frequent comorbidity [11], since its prevalence is estimated at 38.0% in this particular subpopulation [12]. However, similar to OSAS [13], there seems to be a special relationship between insomnia disorder and CVD. Indeed, insomnia disorder is a frequent comorbidity in individuals with CVD, and the prevalence of CVD is not negligible in individuals with insomnia disorder [14,15]. In addition, insomnia disorder appears to promote higher cardiovascular morbidity and mortality both in the general population and in individuals with CVD [16]. Nevertheless, this negative impact of insomnia disorder on cardiovascular prognosis seems to be only associated with some specific subtypes of this disorder [17]. Indeed, some studies have shown that insomnia sufferers with short sleep duration presented a higher risk of CVD than those without short sleep duration [17]. However, despite this high prevalence of comorbid insomnia disorder in apnoeic individuals, and its potential involvement in the occurrence of CVD, the potential role played by this disorder and its specific subtypes in the 10-year CVD risk has been poorly studied in this particular subpopulation [18]. Indeed, most of the studies available in the literature have mainly investigated the impact of comorbid insomnia disorder on the occurrence of some conventional cardiovascular risk factors and some specific CVD in apnoeic individuals [19,20,21]. Thus, given the limited data available in the literature, it could be interesting to study the 10-year CVD risk associated with comorbid insomnia disorder and its specific subtypes in apnoeic individuals in order to better understand the poor cardiovascular prognosis of this particular subpopulation.

The aim of this study was, therefore, to empirically investigate the 10-year CVD risk associated with comorbid insomnia disorder and its specific subtypes in apnoeic individuals. Our hypothesis was that comorbid insomnia disorder and, more specifically, its subtype with short sleep duration are associated with higher 10-year CVD risk in apnoeic individuals. The objective of this approach was to provide healthcare professionals caring for apnoeic individuals with reliable data regarding the 10-year CVD risk associated with comorbid insomnia disorder and its specific subtypes in order to allow the development of more targeted cardiovascular prevention strategies in this particular subpopulation.

## 2. Materials and Methods

### 2.1. Population

A total of 1104 apnoeic individuals were recruited from the clinical database of the Erasme Hospital Sleep Laboratory, which contains demographic and polysomnographic data from individuals who performed a polysomnographic recording between 2002 and 2019. The inclusion and exclusion criteria applied for the recruitment of these 1104 apnoeic individuals are available in Table 1 [22]. In this study, we included only apnoeic individuals because our objective was to focus on this particular subpopulation, in which the occurrence of comorbid insomnia disorder may negatively impact the cardiovascular prognosis [18]. Finally, the detailed description of the outpatient recruitment procedure for the apnoeic patients included in this study is available in Supplementary Materials—Section S1 [23].

### 2.2. Method

#### 2.2.1. Medical and Psychiatric Assessment of Participants

During their admission to the Sleep Laboratory, all apnoeic individuals included in this study benefited from a medical interview and a somatic check-up (including blood test, electrocardiogram, daytime electroencephalogram, and urine analysis) in order to allow a systematic diagnosis of their potential somatic comorbidities. Following this comprehensive somatic assessment, a systematic diagnosis of conventional cardiovascular risk factors (type 2 diabetes (American Diabetes Association diagnostic criteria), hypertension (World Health Organization diagnostic criteria), dyslipidemia (International Diabetes Federation diagnostic criteria), and cardiovascular comorbidities) was performed for all apnoeic individuals included in this study (a detailed description of the diagnostic criteria used is available in Supplementary Materials—Section S2) [24,25,26,27].

Based on these different elements collected during this systematic somatic assessment, the Framingham Risk Score was used to estimate the 10-year risk of manifesting clinical CVD (cardiovascular mortality, coronary heart disease, cerebrovascular disease, peripheral arterial disease, or heart failure) in apnoeic individuals included in this study [28]. The prediction model of the Framingham Risk Score integrates age, sex, smoking status, systolic blood pressure, taking antihypertensive medication, total-cholesterol levels, HDL-cholesterol levels, and diabetes status [28]. A Framingham Risk Score <10% indicates a low 10-year CVD risk, whereas a Framingham Risk Score ≥10% indicates a moderate-to-high 10-year CVD risk [28]. Finally, the Framingham Risk Score is a cardiovascular risk score frequently used in the subpopulation of apnoeic individuals [18,29,30].

After this comprehensive somatic assessment, all apnoeic individuals included in this study benefited from a systematic psychiatric evaluation by a unit psychiatrist to diagnose their potential psychiatric comorbidities according to the diagnostic criteria of the DSM-IV-TR (before 2013) or DSM 5 (after 2013) [31,32].

Finally, all apnoeic individuals included in this study completed a series of self-questionnaires during their admission to the Sleep Laboratory to allow a first assessment of their subjective complaints of depression (Beck Depression Inventory (reduced to 13 items)), insomnia (Insomnia Severity Index), and daytime sleepiness (Epworth Sleepiness Scale) (a detailed description is available in Supplementary Materials—Section S3) [27].

#### 2.2.2. Sleep Evaluation and Study

During their admission to the Sleep Laboratory, all apnoeic individuals included in this study benefited from a specific sleep interview by a unit psychiatrist to allow a systematic assessment of their sleep-related complaints, including sleeping habits, symptoms of insomnia disorders, symptoms of sleep-related breathing disorders, symptoms of central hypersomnia, symptoms of circadian rhythm sleep–wake disorders, symptoms of parasomnias, symptoms of restless leg syndrome (RLS), and abnormal nocturnal movements (such as periodic limb movements during sleep (PLMs)).

The participants benefited from a polysomnographic recording from which the data were collected for the analyses. These polysomnographic recordings performed in the Sleep Laboratory (accredited by the Belgian National Institute for Health and Disability Insurance for the diagnosis and treatment of OSAS) meet the recommendations of the American Academy of Sleep Medicine [33]. The detailed description of the stay conditions at the Sleep Laboratory and the applied polysomnography-montage are available in Supplementary Materials—Section S4 [23,27]. Finally, under the supervision of certified somnologists, these polysomnographic recordings were visually scored by specialised technicians according to the criteria of the American Academy of Sleep Medicine [34,35].

Through these different steps, all apnoeic individuals included in this study benefited from an assessment of their OSAS severity—mild (apnoea–hypopnoea index ≥5/hour and <15/hour), moderate (obstructive apnoea–hypopnoea index ≥15/hour and <30/hour), and severe (obstructive apnoea–hypopnoea index ≥30/hour), and a systematic diagnosis of their potential comorbid sleep disorder—moderate-to-severe PLMs (PLMs index was ≥15/hour), RLS (International Restless Legs Syndrome Study Group diagnostic criteria), insomnia disorder (American Academy of Sleep Medicine Work Group diagnostic criteria), and short sleep duration (<6 h) [36,37,38,39,40].

### 2.3. Statistical Analyses

Statistical analyses were performed using Stata 14. The normal distribution of the data was verified using histograms, boxplots, and quantile-quantile plots, and the equality of variances was checked using Levene’s test.

We divided our sample of apnoeic individuals into a control group at low 10-year CVD risk and a patient group at moderate-to-high 10-year CVD risk. Only apnoeic individuals with a Framingham Risk Score ≥10% were included in the patient group at moderate-to-high 10-year CVD risk [28].

Given the asymmetric distribution of most continuous variables, non-parametric tests (Wilcoxon test) based on the medians (P25–P75) were used to demonstrate significant differences between the different groups of apnoeic individuals. Regarding categorical variables, percentages were used for descriptive analyses, and Chi^2^ tests were used for comparative analyses.

Univariate logistic regression models were used to study the 10-year CVD risk associated with comorbid insomnia complaints (categorised: no, short sleep duration alone, comorbid insomnia disorder), comorbid insomnia subtypes (categorised: no, short sleep duration alone, comorbid insomnia disorder without short sleep duration, comorbid insomnia disorder with short sleep duration), and the potential confounding factors (detailed description available in Supplementary Materials—Section S5). 

In the multivariate logistic regression models, the 10-year CVD risk associated with comorbid insomnia complaints and comorbid insomnia subtypes was only adjusted for significant confounding factors during the univariate analyses. The adequacy of these different models was verified by the Hosmer and Lemeshow test, whereas the specificity of the model was verified by the Link test.

The results were considered significant when the *p*-value was <0.05.

## 3. Results

### 3.1. Polysomnographic Data 

Compared to those with low 10-year CVD risk, apnoeic individuals with moderate-to-high 10-year CVD risk showed:

- Reductions in sleep efficiency, sleep period time, total sleep time, % slow-wave sleep, and % REM sleep (Table 2).
- Increases in % stage 1, % wake after sleep onset, micro-arousal index, obstructive apnoea–hypopnoea index, oxygen desaturation index, total time under 90% of Sao2, and PLMs index (Table 2).

There were no significant differences between the two groups for sleep latency, % stage 2, REM latency, or number of awakenings (Table 2).

### 3.2. Demographic Data 

Moderate-to-high 10-year CVD risk was present in 59.6% (*n* = 658) of apnoeic individuals from our sample (Table 3). Male sex, body mass index ≥25 and <30 kg/m^2^, body mass index ≥30 kg/m^2^, age ≥54 years, smoking, alcohol consumption, moderate-to-severe OSAS, RLS alone or combined with PLMs, short sleep duration alone, comorbid insomnia disorder, type 2 diabetes, untreated hypertension, controlled hypertension, uncontrolled hypertension, dyslipidaemia without statin therapy, dyslipidaemia with statin therapy, cardiovascular comorbidities, aspirin therapy, and CRP levels ≥1 mg/L were more frequent in apnoeic individuals with moderate-to-high 10-year CVD risk than in those with low 10-year CVD risk (Table 3). In addition, apnoeic individuals with moderate-to-high 10-year CVD risk had higher body mass index, age, and CRP levels than those with low 10-year CVD risk (Table 3). There were no significant differences between the two groups for snoring, excessive daytime sleepiness, depression status, Epworth Sleepiness Scale scores, Beck Depression Inventory scores, and Insomnia Severity Index scores (Table 3). Finally, in apnoeic individuals, comorbid insomnia disorder was very frequent, since its prevalence was 40.0% in this particular subpopulation (Table 3).

### 3.3. Univariate Analyses for 10-Year CVD Risk Associated with Comorbid Insomnia Complaints and Potential Confounding Factors in Apnoeic Individuals 

Male sex, overweight, obesity, older age, smoking, alcohol consumption, moderate-to-severe OSAS, RLS alone or combined with PLMs, short sleep duration alone, comorbid insomnia disorder, type 2 diabetes, untreated hypertension, controlled hypertension, uncontrolled hypertension, dyslipidaemia without statin therapy, dyslipidaemia with statin therapy, cardiovascular comorbidities, aspirin therapy, and CRP levels ≥1 mg/L were significantly associated with moderate-to-high 10-year CVD risk in apnoeic individuals (Table 4).

### 3.4. Multivariate Analyses for 10-Year CVD Risk Associated with Comorbid Insomnia Complaints in Apnoeic Individuals 

After adjustment for the main confounding factors associated with cardiovascular risk highlighted during the univariate analyses, multivariate logistic regression analyses revealed that unlike short sleep duration alone, only comorbid insomnia disorder was significantly associated with moderate-to-high 10-year CVD risk in apnoeic individuals (Table 5).

### 3.5. Additional Univariate and Multivariate Analyses for 10-Year CVD Risk Associated with Comorbid Insomnia Subtypes in Apnoeic Individuals

Unlike comorbid insomnia disorder without short sleep duration, only short sleep duration alone and comorbid insomnia disorder with short sleep duration were more frequent in apnoeic individuals with moderate-to-high 10-year CVD risk than in those with low 10-year CVD risk (Table 6). In addition, during univariate logistic regression analyses, unlike comorbid insomnia disorder without short sleep duration, only short sleep duration alone and comorbid insomnia disorder with short sleep duration were significantly associated with moderate-to-high 10-year CVD risk in apnoeic individuals (Table 6). Finally, after adjustment for the main confounding factors associated with cardiovascular risk highlighted during the univariate analyses, multivariate logistic regression analyses revealed that unlike short sleep duration alone and comorbid insomnia disorder without short sleep duration, only comorbid insomnia disorder with short sleep duration was significantly associated with moderate-to-high 10-year CVD risk in apnoeic individuals (Table 6).

## 4. Discussion

In this study, we demonstrated that 59.6% of apnoeic individuals had a moderate-to-high 10-year CVD risk, which is significantly higher than in the general population [41]. However, the rate of apnoeic individuals with moderate-to-high 10-year CVD risk highlighted in our study seems to be higher than that of the study by Li et al. (2020) [42]. Indeed, in this previous study, only 34.0% of apnoeic individuals had a moderate-to-high 10-year CVD risk [42]. However, compared to our study, the apnoeic individuals recruited in the study by Li et al. (2020) had a better demographic (younger age and lower body mass index) and cardiometabolic profile (lower prevalence of type 2 diabetes, hypertension, and dyslipidaemia) [42], which may have led to an underestimation of the 10-year CVD risk in their study given the major role played by these demographic and cardiometabolic factors in the development of CVD [28]. On the other hand, the rate of apnoeic individuals with moderate-to-high 10-year CVD risk demonstrated in our study seems to be smaller than that of the study by Luyster et al. (2014) (66.4%) [18]. However, unlike our study, where OSAS was diagnosed during polysomnographic recordings, the use of the Multivariable Apnoea Prediction Questionnaire to identify individuals at high risk of OSAS could explain the overestimation of the 10-year CVD risk in their study given that the algorithm of this screening tool seems to favour the recruitment of individuals with higher cardiovascular risk (higher body mass index, older age, and more severe cardiometabolic comorbidities) in the groups at high risk of OSAS [43,44]. Finally, the rate of apnoeic individuals with moderate-to-high 10-year CVD risk from our study seems to be consistent with that of the studies by Matthews et al. (2011) and Cao et al. (2022) (57.1%), which recruited apnoeic individuals with demographic and cardiometabolic features more similar to those of our sample of apnoeic individuals [29,30]. Thus, regardless of some methodological differences with other studies available in the literature, we have confirmed that apnoeic individuals are a subpopulation at high risk of CVD, which justifies a better identification of the cardiovascular risk factors specific to this particular subpopulation.

Similar to the data available in the literature [12], we confirmed that comorbid insomnia disorder is common in apnoeic individuals. Indeed, in our study, 40% of apnoeic individuals had comorbid insomnia disorder, which highlights the importance of the co-occurrence of insomnia disorder and OSAS. In addition, we have shown that comorbid insomnia disorder and, more particularly, its subtype with short sleep duration were significantly associated with moderate-to-high 10-year CVD risk in apnoeic individuals. Pathophysiologically, several elements could help to better understand this frequent occurrence of comorbid insomnia disorder and its potential involvement in the 10-year CVD risk in apnoeic individuals. First, repeated nocturnal awakenings related to OSAS may induce the development of psychophysiological conditioning processes promoting dysfunctional sleep behaviours [45]. However, since dysfunctional sleep behaviours are one of the main pathophysiological mechanisms involved in the acute onset and maintenance of insomnia disorder [46], the development of these dysfunctional sleep behaviours related to OSAS could explain the frequent co-occurrence of insomnia disorder in our sample of apnoeic individuals. Secondly, in the literature, there are arguments in support of a potential synergistic effect of the co-occurrence between insomnia disorder and OSAS on some pathophysiological mechanisms (deregulation of the hypothalamic–pituitary–adrenal axis, hyperactivation of the sympathetic nervous system, and activation of pro-inflammatory mechanisms) [47,48]. However, since these pathophysiological mechanisms play a central role in the development of CVD in both apnoeic and insomniac individuals [6,49], the potential negative cumulative effect on the cardiovascular outcome of this pathophysiological synergy between insomnia disorder and OSAS could explain the higher 10-year CVD risk associated with comorbid insomnia disorder highlighted in our sample of apnoeic individuals. Third, in our study, we found that this higher 10-year CVD risk associated with comorbid insomnia disorder in apnoeic individuals appears to be mediated by sleep duration. Indeed, unlike comorbid insomnia disorder without short sleep duration, only comorbid insomnia disorder with short sleep duration was significantly associated with moderate-to-high 10-year CVD risk in apnoeic individuals. However, in insomnia sufferers with short sleep duration, the pathophysiological mechanisms favouring the development of CVD are more marked than in insomnia sufferers without short sleep duration [50], which could potentially explain this mediating effect of sleep duration on the 10-year CVD risk associated with comorbid insomnia disorder demonstrated in our sample of apnoeic individuals. Thus, based on these different elements, it seems essential to systematically screen and adequately treat comorbid insomnia disorder and, more particularly, its subtype with short sleep duration in apnoeic individuals in order to allow better cardiovascular prevention in this particular subpopulation.

The demonstration of this higher 10-year CVD risk associated with comorbid insomnia disorder and, more particularly, its subtype with short sleep duration in apnoeic individuals could allow a better understanding of the limited effect of OSAS treatments on reducing cardiovascular risk in this particular subpopulation [51]. Indeed, in apnoeic individuals, the absence of appropriate management of comorbid insomnia disorder could induce the persistence of pathophysiological mechanisms favouring the emergence of CVD [16], both by the direct negative effect of insomnia disorder on cardiovascular outcome and by the indirect negative effect of insomnia disorder on compliance with OSAS treatments [52,53]. However, although the implementation of an adequate combined treatment of comorbid insomnia disorder could open new perspectives to allow a better cardiovascular outcome in apnoeic individuals [54], it seems essential to take into account the specific features of this particular subpopulation for the choice of this combined treatment in order to avoid the establishment of treatments with a negative impact for the management of OSAS [55]. Indeed, since most pharmacological treatments for comorbid insomnia disorder may have a deleterious effect on respiratory parameters in apnoeic individuals, cognitive–behavioural therapy for insomnia combined with optimal treatment of OSAS (lifestyle changes plus continuous positive airway pressure therapy/mandibular advancement devices/surgery) seems to be the best therapeutic option for apnoeic individuals with comorbid insomnia disorder [56,57]. Finally, alongside this combined treatment of comorbid insomnia disorder in apnoeic individuals, it is essential to establish adequate therapeutic strategies for conventional cardiovascular risk factors in order to allow integrated cardiovascular management in this particular subpopulation [58].

### Limitations

The results obtained in our study come from retrospective data that, even if they have been encoded in a systematic manner, cannot be verified directly with the subject in most cases, which means that our results need to be replicated in prospective studies. Furthermore, we only focused on OSAS, which means that our results cannot be generalised to other types of sleep-related breathing disorders (such as central sleep apnoea, sleep-related hypoventilation, or sleep-related hypoxemia disorder). In addition, although the Framingham Risk Score is a cardiovascular risk score frequently used in apnoeic individuals, it only allows an indirect measurement of the 10-year CVD risk, which may potentially limit the interpretation of our results. Finally, our database only contains apnoeic individuals who had agreed to undergo a Sleep Laboratory evaluation, which may also limit the generalisability of our results.

## 5. Conclusions

In this study, we confirmed that insomnia disorder was a frequent comorbidity in apnoeic individuals. Indeed, the prevalence of comorbid insomnia disorder was 40.0% in our sample of apnoeic individuals. In addition, we demonstrated a moderate-to-high 10-year CVD risk in 59.6% of apnoeic individuals from our sample, which confirms that apnoeic individuals are a subpopulation at high risk of CVD. Finally, we highlighted that comorbid insomnia disorder and, more specifically, its subtype with short sleep duration appear to have a negative cumulative effect on 10-year CVD risk in apnoeic individuals, which justifies more systematic research and adequate therapeutic management of this disorder in order to allow for better cardiovascular prevention in this particular subpopulation.

## Figures and Tables

**Table 1:** Inclusion and exclusion criteria.

Inclusion Criteria	Exclusion Criteria
Age ≥ 40 years	Severe psychiatric disorder Psychotic disorder Bipolar disorder Current or past substance abuse
OSAS according to the diagnostic criteria of the American Academy of Sleep Medicine [22]	Uncontrolled somatic disorder Chronic hepatic disorder Chronic pancreatic disorder Chronic pulmonary disorder Chronic cardiovascular disorder Chronic renal disorder Autoimmune disorder Infectious or inflammatory disorder Disorder altering the activity of the hypothalamic–pituitary–adrenal axis
Absence of previous CVD Coronary heart disease Cerebrovascular disease Peripheral arterial disease Heart failure	Sleep disorder Central hypersomnia Parasomnia Predominantly central sleep apnoea syndrome OSAS being treated before the sleep examination
	Lesions or malformations Current or past cranial trauma Current or past central nervous system lesions involving the respiratory centres Craniofacial or thoracic cavity malformations
	Pregnancy

CVD = cardiovascular disease, OSAS = obstructive sleep apnoea syndrome.

**Table 2:** Polysomnographic data (*n* = 1104).

	Whole Sample (*n* = 1104)	Subjects with Low 10-Year CVD Risk (*n* = 446)	Subjects with Moderate-to-High 10-Year CVD Risk (*n* = 658)	*p*-Value
Sleep latency (min)	25.0 (12.7–50.5)	23.2 (12.0–49.0)	26.6 (13.0–52.0)	0.154
Sleep efficiency (%)	77.0 (67.4–83.9)	79.4 (71.1–85.8)	75.0 (64.9–82.8)	<0.001
Sleep period time (min)	452.5 (414.4–485.3)	457.8 (422.0–492.0)	451.0 (407.0–482.0)	0.005
Total sleep time (min)	379.0 (332.8–422.3)	396.2 (348.7–430.7)	368.0 (321.0–413.3)	<0.001
% stage 1	9.0 (6.0–13.0)	8.0 (5.8–11.8)	9.3 (6.8–13.8)	<0.001
% stage 2	54.0 (47.1–60.0)	54.0 (48.0–59.6)	54.0 (46.0–60.0)	0.347
% slow-wave sleep	1.8 (0.0–7.0)	3.5 (0.4–9.0)	1.0 (0.0–5.3)	<0.001
% REM sleep	15.2 (11.0–19.7)	16.2 (12.4–20.3)	14.9 (10.0–19.0)	<0.001
REM latency (min)	83.5 (59.0–124.0)	85.0 (61.0–121.3)	81.5 (57.5–129.0)	0.631
% wake after sleep onset	14.6 (8.8–23.0)	12.9 (7.8–19.5)	15.4 (9.8–24.1)	<0.001
Number of awakenings	35 (24–52)	34 (23–49)	36 (25–53)	0.169
Micro-arousal index	17 (11–31)	15 (10–23)	19 (12–32)	<0.001
Apnoea–hypopnoea index	14 (8–30)	12 (7–22)	17 (9–35)	<0.001
Oxygen desaturation index	5 (2–14)	4 (1–8)	6 (2–18)	<0.001
Total time under 90% of SaO2 (min)	14.6 (1.7–69.0)	6.0 (0.5–32.5)	21.5 (3.5–96.0)	<0.001
PLMs index	2 (0–11)	1 (0–8)	3 (0–14)	0.001
	**Median (P25–P75)**	**Median (P25–P75)**	**Median (P25–P75)**	**Wilcoxon Test**

CVD = cardiovascular disease, OSAS = obstructive sleep apnoea syndrome, PLMs = periodic limb movements during sleep, REM = rapid eye movement.

**Table 3:** Sample description (*n* = 1104).

Variables	Categories	%	Subjects with Low 10-Year CVD Risk	Subjects with Moderate-to-High 10-Year CVD Risk	*p*-ValueChi^2^
Gender	Female (*n* = 263) Male (*n* = 841)	23.8% 76.2%	65.0% 32.7%	35.0% 67.3%	<0.001
BMI (kg/m^2^)	<25 (*n* = 194) ≥25 and <30 (*n* = 432) ≥30 (*n* = 478)	17.6% 39.1% 43.3%	55.7% 39.8% 34.7%	44.3% 60.2% 65.3%	<0.001
Age (years)	<54 (*n* = 535) ≥54 (*n* = 569)	48.5% 51.5%	57.4% 24.4%	42.6% 75.6%	<0.001
Smoking	No (*n* = 900) Yes (*n* = 204)	81.5% 18.5%	45.8% 16.7%	54.2% 83.3%	<0.001
Alcohol	No (*n* = 725) Yes (*n* = 379)	65.7% 34.3%	42.6% 36.2%	57.4% 63.8%	0.037
Snoring	Non (*n* = 173) Yes (*n* = 931)	15.7% 84.3%	38.7% 40.7%	61.3% 59.3%	0.626
OSAS severity	Mild (*n* = 559) Moderate (*n* = 269) Severe (*n* = 276)	50.6% 24.4% 25.0%	49.4% 35.7% 26.8%	50.6% 64.3% 73.2%	<0.001
Sleep movement disorders	No (*n* = 861) Moderate-to-severe PLMs alone (*n* = 70) RLS alone or combined with PLMs (*n* = 173)	78.0% 6.3% 15.7%	42.6% 38.6% 30.1%	57.4% 61.4% 69.9%	0.008
Comorbid insomnia complaints	No (*n* = 441) Short sleep duration alone (*n* = 221) Comorbid insomnia disorder (*n* = 442)	39.9% 20.1% 40.0%	46.0% 32.1% 38.9%	54.0% 67.9% 61.1%	0.002
Excessive daytime sleepiness	No (*n* = 649) Yes (*n* = 455)	58.8% 41.2%	38.4% 43.3%	61.6% 56.7%	0.100
Type 2 diabetes	No (*n* = 890) Yes (*n* = 214)	80.6% 19.4%	48.1% 8.4%	51.9% 91.6%	<0.001
Hypertension	No (*n* = 496) Untreated (*n* = 169) Controlled (*n* = 301) Uncontrolled (*n* = 138)	44.9% 15.3% 27.3% 12.5%	60.1% 29.6% 28.6% 8.7%	39.9% 70.4% 71.4% 91.3%	<0.001
Dyslipidaemia	No (*n* = 460) Without statin therapy (*n* = 366) With statin therapy (*n* = 278)	41.7% 33.1% 25.2%	56.1% 32.2% 25.2%	43.9% 67.8% 74.8%	<0.001
Cardiovascular comorbidities	No (*n* = 945) Yes (*n* = 159)	85.6% 14.4%	42.3% 28.9%	57.7% 71.1%	0.001
Aspirin therapy	No (*n* = 913) Yes (*n* = 191)	82.7% 17.3%	45.1% 17.8%	54.9% 82.2%	<0.001
CRP (mg/L)	<1 (*n* = 294) ≥1 (*n* = 810)	26.6% 73.4%	49.3% 37.2%	50.7% 62.8%	<0.001
Depression	No (*n* = 649) Remitted (*n* = 208) Current (*n* = 247)	58.8% 18.8% 22.4%	39.8% 44.7% 38.5%	60.2% 55.3% 61.5%	0.349
10-year CVD Risk	Low (*n* = 446) Moderate-to-high (*n* = 658)	40.4% 59.6%			
	**Median** **(P25–P75)**				**Wilcoxon Test**
BMI (kg/m^2^)	29.0 (26.1–32.9)		28.1 (25.1–31.7)	29.6 (26.8–33.6)	<0.001
Age (years)	54 (48–61)		49 (45–55)	57 (52–63)	<0.001
ESS	9 (6–13)		9 (6–13)	9 (6–12)	0.397
BDI	3 (1–7)		3 (1–7)	3 (2–7)	0.415
ISI	13 (8–17)		13 (8–17)	13 (9–17)	0.846
CRP (mg/L)	1.7 (1.0–3.6)		2.2 (1.0–6.9)	2.9 (1.2–7.7)	0.030
Framingham Risk Score (%)	11.9 (7.2–19.7)		6.3 (4.6–8.2)	17.9 (13.0–25.2)	<0.001

CVD = cardiovascular disease, OSAS = obstructive sleep apnoea syndrome, BMI = body mass index, CRP = C-reactive protein, PLMs = periodic limb movements during sleep, RLS = restless legs syndrome, ESS = Epworth Sleepiness Scale, BDI = Beck Depression Inventory, ISI = Insomnia Severity Index.

**Table 4:** Univariate analyses for 10-year CVD risk associated with comorbid insomnia complaints and potential confounding factors in apnoeic individuals (*n* = 1104).

Variables	OR (CI 95%)	*p*-Value
Gender		<0.001
Female Male	1 3.83 (2.86 to 5.12)	
BMI (kg/m^2^)		<0.001
<25	1	
≥25 and <30	1.90 (1.35 to 2.67)	
≥30	2.36 (1.68 to 3.32)	
Age (years)		<0.001
<54	1	
≥54	4.16 (3.22 to 5.38)	
Smoking		<0.001
No	1	
Yes	4.22 (2.86 to 6.24)	
Alcohol		0.038
No	1	
Yes	1.31 (1.02 to 1.70)	
Snoring		0.626
No	1	
Yes	0.92 (0.66 to 1.28)	
OSAS severity		<0.001
Mild	1	
Moderate	1.76 (1.30 to 2.37)	
Severe	2.66 (1.95 to 3.64)	
Sleep movement disorders		0.009
No	1	
Moderate-to-severe PLMs	1.18 (0.72 to 1.95)	
RLS alone or combined with PLMs	1.73 (1.22 to 2.46)	
Comorbid insomnia complaints		0.002
No	1	
Short sleep duration alone	1.80 (1.28 to 2.53)	
Comorbid insomnia disorder	1.34 (1.02 to 1.75)	
Excessive daytime sleepiness		0.101
No	1	
Yes	0.82 (0.64 to 1.04)	
Type 2 diabetes		<0.001
No	1	
Yes	10.08 (6.12 to 16.64)	
Hypertension		<0.001
No	1	
Untreated	3.58 (2.46 to 5.22)	
Controlled	3.76 (2.77 to 5.12)	
Uncontrolled	15.80 (8.51 to 29.34)	
Dyslipidaemia		<0.001
No	1	
Without statin therapy	2.68 (2.02 to 3.57)	
With statin therapy	3.80 (2.74 to 5.27)	
Cardiovascular comorbidities		0.002
No	1	
Yes	1.80 (1.25 to 2.60)	
Aspirin therapy		<0.001
No	1	
Yes	3.80 (2.56 to 5.63)	
CRP (mg/L)		<0.001
<1	1	
≥1	1.65 (1.26 to 2.15)	
Depression		0.35
Non	1	
Remitted	0.82 (0.60 to 1.12)	
Current	1.06 (0.78 to 1.43)	

OSAS = obstructive sleep apnoea syndrome, BMI = body mass index, CRP = C-reactive protein, PLMs = periodic limb movements during sleep, RLS = restless legs syndrome.

**Table 5:** Multivariate analyses for 10-year CVD risk associated with comorbid insomnia complaints in apnoeic individuals (*n* = 1104).

Variables	OR Adjusted (CI 95%)	*p*-Value
Comorbid insomnia complaints		0.037
No	1	
Short sleep duration alone	0.99 (0.60 to 1.65)	
Comorbid insomnia disorder	1.64 (1.09 to 2.45)	

Model adjusted for gender, BMI, age, smoking, alcohol, OSAS severity, sleep movement disorders, type 2 diabetes, hypertension, dyslipidaemia, cardiovascular comorbidities, aspirin therapy, and CRP levels.

**Table 6:** Additional univariate and multivariate analyses for 10-year CVD risk associated with comorbid insomnia subtypes in apnoeic individuals (*n* = 1104).

Variables	%	Subjects with Low 10-Year CVD Risk	Subjects with Moderate-to-High 10-Year CVD Risk	Model 1 OR Unadjusted (CI 95%)	*p*-Value	Model 2 OR Adjusted (CI 95%)	*p*-Value
Comorbid insomnia subtypes					<0.001		0.018
No	39.9% (*n* = 441)	46.0%	54.0%	1		1	
Short sleep duration alone	20.1% (*n* = 221)	32.1%	67.9%	1.80 (1.28 to 2.53)		0.99 (0.60 to 1.65)	
Without short sleep duration	22.4% (*n* = 247)	44.9%	55.1%	1.05 (0.76 to 1.43)		1.26 (0.78 to 2.04)	
With short sleep duration	17.6% (*n* = 195)	31.3%	68.7%	1.87 (1.31 to 2.67)		2.22 (1.33 to 3.72)	

Model 1 = model unadjusted. Model 2 = model adjusted for gender, BMI, age, smoking, alcohol, OSAS severity, sleep movement disorders, type 2 diabetes, hypertension, dyslipidaemia, cardiovascular comorbidities, aspirin therapy, and CRP levels. CVD = cardiovascular disease.

## Data Availability

The datasets used and/or analysed during the current study are available from the corresponding author on reasonable request.

## Supplementary Materials

The following supporting information can be downloaded at: https://www.mdpi.com/article/10.3390/life12070944/s1, Section S1 Detailed description of the outpatient recruitment procedure for the apnoeic patients included in this study; Section S2: Detailed description of the diagnostic criteria used for the conventional cardiovascular risk factors; Section S3: Detailed description of self-questionnaires used; Section S4: Description of the stay conditions at the Sleep Laboratory and description of the applied polysomnography-montage; Section S5: Description of the confounding factors included in the univariate analyses. References [24,25,26,59,60,61,62,63,64,65,66,67,68] are cited in the Supplementary Materials.

## Abbreviations

CRP: C-Reactive Protein; CVD: Cardiovascular Disease; DSM: Diagnostic and Statistical Manual of Mental Disorders; OSAS: Obstructive Sleep Apnoea Syndrome; PLMs: Periodic Limb Movements during Sleep; REM: Rapid Eye Movement; RLS: Restless Legs Syndrome.
