# Supplementary material for: Risk of Cardiovascular Disease in Apnoeic Individuals: Role of Comorbid Insomnia Disorder
Source: Life (Basel). 2022 Jun 23;12(7):944. doi: 10.3390/life12070944 (PMC9319904; doi:10.3390/life12070944)
Supplement: Supplementary file 1 [file life-12-00944-s001.zip › life-1695646-supplementary.pdf]

Supplementary Data

Section S1

Detailed description of the outpatient recruitment procedure for the apnoeic patients included in this study

These apnoeic individuals were referred to the sleep laboratory by physicians specialised in sleep medicine after an outpatient consultation during which a preliminary assessment of their complaints related to sleep, their ongoing psychotropic/somatic treatments and their somatic/psychiatric comorbidities was systematically performed in order to allow a first diagnostic hypothesis. These polysomnographic examinations were performed in these apnoeic individuals to allow an objective assessment of their sleep complaints and confirm the suspicion of OSAS highlighted during the outpatient assessment.

Section S2Detailed description of the diagnostic criteria used for the conventional cardiovascular risk factors

In all apnoeic individuals included in this study, conventional cardiovascular risk factors were considered present according to the following criteria:

- Type 2 diabetes (*American Diabetes Association* diagnostic criteria): glycated haemoglobin (HbA1c) ≥6.5% or fasting plasma glucose ≥126mg/dl or two-hour plasma glucose ≥200 mg/dl during an oral glucose tolerance test or random plasma glucose ≥200 mg/dl in patients with classic symptoms of hyperglycaemia or self-reported diagnosis of clinically demonstrated type 2 diabetes or taking antidiabetic medication [24]. In the absence of unequivocal hyperglycaemia, criteria 1–3 should be confirmed by repeat testing. In addition, diabetes must have begun in adulthood [24].
- Hypertension (*World Health Organization* diagnostic criteria): mean systolic blood pressure ≥140 mmHg or mean diastolic blood pressure ≥90 mmHg or self-reported diagnosis of clinically demonstrated hypertension or use of antihypertensive medication [25]. In treated hypertensive individuals, controlled hypertension was defined as the presence of mean systolic blood pressure <140 mmHg and mean diastolic blood pressure <90 mmHg whereas uncontrolled hypertension was defined as the presence of mean systolic blood pressure ≥140 mmHg and/or mean diastolic blood pressure ≥90 mmHg [25].
- Dyslipidemia (*International Diabetes Federation* diagnostic criteria): plasma triglyceride level ≥150 mg/dl or plasma HDL-cholesterol level <40 mg/dl for men or plasma HDL-cholesterol level <50 mg/dl for women or use of treatment for dyslipidaemia [26].

- Cardiovascular comorbidities (excluding hypertension): presence of one or more comorbid cardiovascular pathologies (cardiac arrhythmias, non-ischemic cardiomyopathy, cardiac valve disease and history of cardiac surgery).

References

24. American Diabetes Association. Diagnosis and classification of diabetes mellitus. *Diabetes Care*. **2014**, *37 Suppl 1*, S81-90.

25. Mills, K.T.; Bundy, J.D.; Kelly, T.N.; Reed, J.E.; Kearney, P.M.; Reynolds, K.; Chen, J.; He, J. Global disparities of hypertension prevalence and control: A systematic analysis of population-based studies from 90 countries. *Circulation*. **2016**, *134(6)*, 441-450.26.

26. Alberti, K.G.; Eckel, R.H.; Grundy, S.M.; Zimmet, P.Z.; Cleeman, J.I.; Donato, K.A.; Fruchart, J.C.; James, W.P.; Loria, C.M.; Smith, S.C. Jr.; International Diabetes Federation Task Force on Epidemiology and Prevention; Hational Heart, Lung, and Blood Institute; American Heart Association; World Heart Federation; International Atherosclerosis Society; International Association for the Study of Obesity. Harmonizing the metabolic syndrome: a joint interim statement of the International Diabetes Federation Task Force on Epidemiology and Prevention; National Heart, Lung, and Blood Institute; American Heart Association; World Heart Federation; International Atherosclerosis Society; and International Association for the Study of Obesity. *Circulation*. **2009**, *120(16)*, 1640-1645.

Section S3 Detailed description of self-questionnaires used

- The Beck Depression Inventory (reduced to 13 items) was used to investigate the presence of depressive symptoms. The 13 items of this scale may be

scored from 0 to 3, which mean that the total score may vary from 0 to 39. A final score of 0-4 indicates an absence of depressive symptoms, 5-7 mild depressive symptoms, 8-15 moderate depressive symptoms, and ≥16 severe depressive symptoms [59].

- The Epworth Sleepiness Scale was used to investigate daytime sleepiness. The 8 items of this scale assessing sleepiness in different daytime situations may be scored from 0 to 3, which mean that the total score may vary from 0 to 24. A final score greater than 10 indicates excessive daytime sleepiness [60].

- The Insomnia Severity Index was used to investigate the severity of insomnia complaints. The 7 items of this index may be scored from 0 to 4, which mean that the total score may vary from 0 to 28. A final score of 0-7 indicates an absence of insomnia complaints, 8-14 subclinical insomnia complaints, 15-21 moderate insomnia complaints, and 22-28 severe insomnia complaints [61].

References

59. Beck, A.T.; Steer, R.A.; Ball, R.; Ranieri, W. Comparison of Beck Depression Inventories -IA and -II in psychiatric outpatients. *J Pers Assess*. **1996**, *67(3)*, 588-597.

60. Johns, M.W. A new method for measuring daytime sleepiness: the Epworth sleepiness scale. *Sleep*. **1991**, *14(6)*, 540-545.

61. Morin, C.M. Insomnia: psychological assessment and management. Guilford Press, New York: 1993.

Section S4 Description of the stay conditions at the Sleep Laboratory

The patients went to bed between 22:00 - 24:00 and got up between 6:00 - 8:00, following their usual schedule. During bedtime hours, the subjects were recumbent and the lights were turned off. Daytime naps were not permitted.

Description of the applied polysomnography-montage

- Two electro-oculogram channels
- Three electroencephalogram channels (Fz-Ax, Cz-Ax and Oz-Ax, where Ax was a A1A2 mastoid reference)
- One submental electromyogram channel
- Electrocardiogram
- Pressure cannula to detect the oro-nasal airflow
- Finger pulse-oximetry
- Microphone to record breathing sounds and snoring
- Plethysmographic inductive belts to measure thoracic and abdominal breathing
- Anterior tibialis electrodes

Section S5 Description of the confounding factors included in the univariate analyses

After a review of the literature on cardiovascular risk factors in apnoeic individuals [62-68], the potential confounding factors included in this study were body mass index (categorised: <25 kg/m², ≥25 & <30 kg/m², ≥30 kg/m²), age (categorised: <54 years, ≥54 years), OSAS severity (categorised: mild, moderate, severe), sleep movement disorders (categorised: no, moderate to severe PLMs, RLS alone or combined with PLMs), hypertension (categorised: absent, untreated, controlled, uncontrolled), dyslipidaemia (categorised: absent, without statin therapy, with statin therapy), CRP levels (categorised: <1 mg/L, ≥1 mg/L), depression (categorised: no, remitted, current) and as binary variables: gender, alcohol consumption, smoking, snoring, excessive daytime sleepiness, type 2 diabetes, cardiovascular comorbidities and aspirin therapy.

References

62. Loke, Y.K.; Brown, J.W.; Kwok, C.S.; Niruban, A.; Myint, P.K. Association of obstructive sleep apnea with risk of serious cardiovascular events: a systematic review and meta-analysis. *Circ Cardiovasc Qual Outcomes*. **2012**, *5(5)*, 720-728.
63. Kendzerska, T.; Gershon, A.S.; Hawker, G.; Leung, R.S.; Tomlinson, G. Obstructive sleep apnea and risk of cardiovascular events and all-cause mortality: a decade-long historical cohort study. *PLoS Med*. **2014**, *11(2)*, e1001599.
64. Kendzerska, T.; Mollayeva, T.; Gershon, A.S.; Leung, R.S.; Hawker, G.; Tomlinson, G. Untreated obstructive sleep apnea and the risk for serious long-term adverse outcomes: a systematic review. *Sleep Med Rev*. **2014**, *18(1)*, 49-59.

65. Bouloukaki, I.; Fanaridis, M.; Stathakis, G.; Ermidou, C.; Kallergis, E.; Moniaki, V.; Mauroudi, E.; Schiza, S.E. Characteristics of Patients with Obstructive Sleep Apnea at High Risk for Cardiovascular Disease. *Medicina (Kaunas)*. **2021**, *57(11)*, 1265.
66. Ayas, N.T.; Hirsch Allen, A.J.; Fox, N.; Peres, B.; Mehrtash, M.; Humphries, K.H.; Jen, R.; Taylor, C.M., van Eeden, S.F. C-Reactive Protein Levels and the Risk of Incident Cardiovascular and Cerebrovascular Events in Patients with Obstructive Sleep Apnea. *Lung*. **2019**, *197(4)*, 459-464.
67. Niiranen, T.J.; Kronholm, E.; Rissanen, H.; Partinen, M.; Jula, A.M. Self-reported obstructive sleep apnea, simple snoring, and various markers of sleep-disordered breathing as predictors of cardiovascular risk. *Sleep Breath*. **2016**, *20(2)*, 589-596.
68. Gottlieb, D.J.; Somers, V.K.; Punjabi, N.M.; Winkelman, J.W. Restless legs syndrome and cardiovascular disease: a research roadmap. *Sleep Med*. **2017**, *31*, 10-17.
